# Effectiveness of Postoperative Single-shot and Continuous Transverse Abdominis Plane Block Compared to Conventional Analgesia in Hand-assisted Laparoscopic Live-donor Nephrectomy

**DOI:** 10.1097/TXD.0000000000001581

**Published:** 2024-02-16

**Authors:** Fransia De Leon, Karima Alghannam, Hadia Lala Gul, Naeem Goussous, Neal Mineyev, Peter A. Than, Richard V. Perez, Junichiro Sageshima

**Affiliations:** 1 School of Medicine, University of California Davis, Sacramento, CA.; 2 Department of Surgery, University of California Davis, Sacramento, CA.; 3 Department of Internal Medicine, University of California Davis, Sacramento, CA.

## Abstract

**Background.:**

Few studies have evaluated the efficacy of transverse abdominis plane (TAP) block in patients undergoing hand-assisted laparoscopic live-donor nephrectomy (HALN). We aimed to evaluate the analgesic effectiveness of TAP block as part of a multimodal pain management regimen in patients undergoing HALN.

**Methods.:**

We retrospectively reviewed the medical records of living kidney donors at our center between June 2016 and February 2020. HALNs were performed via a transperitoneal approach through a suprapubic incision. Additional laparoscopic ports were used in the upper midabdomen. In consenting donors, TAP block was performed postoperatively under ultrasound guidance with either a single-shot or continuous infusion of long-acting local anesthetic (0.2%–0.5% ropivacaine). All the patients received postoperative around-the-clock ketorolac and acetaminophen.

**Results.:**

Overall, 72 donors received the block (block group, 38 single-shot, 34 continuous), whereas 86 donors did not receive the block (control group). Baseline characteristics were comparable between the groups except for body weight (control: 71.8 ± 13.3 versus block: 77.8 ± 17.3 kg; *P* = 0.01) and intraoperative opioid dose (32.1 ± 9.6 versus 26.6 ± 10.7 morphine milligram equivalents; *P* < 0.001). After adjusting for baseline differences, postoperative opioid requirements were similar between the groups. When the baseline pain scale was adjusted for, there was no difference in the overall pain scale scores between the groups (*P* = 0.242). Subgroup analyses comparing single-shot or continuous TAP versus control did not show any differences.

**Conclusions.:**

With the caveat of the retrospective nature of the study, the adjunctive effect of TAP block after transabdominal HALN was limited when other multimodal analgesia was used.

The Enhanced Recovery After Surgery (ERAS) model has popularized and improved post-surgical patient outcomes as providers shift toward opioid-sparing analgesia.^1^ In addition, implementation of opioid-sparing regimens is an important strategy in responding to the opioid crisis, aimed at reducing the risk of long-term opioid dependence in postoperative patients. Thus, regional analgesic techniques in transplant donors and recipients are preferred in perioperative regimens.^2^ Living kidney donation is a crucial organ source for patients with end-stage kidney disease. A live-donor kidney transplant offers patients better graft survival and long-term life expectancy compare to deceased donor transplantation.^3^ As donors are relatively healthy individuals who undergo surgery that does not provide them a direct medical benefit, improving post-surgical outcomes and minimizing postoperative pain and potential risks to the remnant kidney are imperative.

Transverse abdominis plane (TAP) block^4,5^ has been shown to lower postoperative narcotic need and pain scores in a range of abdominal surgeries,^6^ including gastric bypass,^7^ laparoscopic cholecystectomy,^8,9^ hernia repairs,^10,11^ and gynecological surgeries.^12-14^ Limited studies, however, have assessed TAP block safety and its adjunctive contribution in an ERAS model or multimodal analgesic regimen for patients undergoing hand-assisted laparoscopic live-donor nephrectomy (HALN).

We aimed to evaluate our center’s experience in the post-surgical recovery and adjunctive role of single-shot and continuous TAP or rectus sheath (RS) block as part of a multimodal pain management regimen for HALN.

## MATERIALS AND METHODS

In this single-center retrospective analysis, we reviewed the medical records of living kidney donors between June 2016 and February 2020. Donors who received a different pain management regimen (from September 2017 to December 2018) due to medication shortage were excluded from the analysis. As markers of accelerated postoperative recovery, we compared postoperative opioid requirements, Visual Analog Pain Scales, and postoperative length of stay (LOS) between donors who received TAP/RS block (block group) and donors who did not receive TAP/RS block (control group). UC Davis Institutional Review Board approved the project.

Anesthesia was induced with propofol, midazolam, and fentanyl and maintained with an inhalation anesthetic and fentanyl/hydromorphone. HALNs were performed via a transperitoneal approach through a suprapubic incision. Two or 3 additional laparoscopic ports were used in the upper midabdomen. The laparoscopic ports received local anesthetic infiltration at the time of insertion.

Preoperatively, anesthesia providers discussed postoperative pain management modalities, and consented donors underwent either a single-shot or continuous TAP/RS block procedure immediately after surgery in the operating room or post-anesthesia care unit (PACU). All block donors received a bolus of long-acting local anesthetic under ultrasound guidance –0.2% to 0.5% ropivacaine (10–50 mL) at the discretion of the anesthesia providers, taking into account individual patient factors, including body weight. In addition, donors with catheter insertion received a continuous infusion of 0.2% ropivacaine (4–12 mL/h), tailed based on the patient’s pain scores and opioid requirement postoperatively. Intraoperative and postoperative narcotic doses were converted to morphine milligram equivalents (MMEs) by using a standard table.

All donors were treated with around-the-clock intravenous ketorolac (15 mg every 6 h) and intravenous and oral acetaminophen (1000 mg every 6 h, reduced to 650–325 mg for smaller patients) as postoperative adjunctive therapy. Intravenous fentanyl and hydromorphone were used for severe or moderate breakthrough pain, respectively, while in the PACU; hydromorphone or morphine was used after PACU discharge. Other adjunctive medications for pain, such as dexamethasone, were not routinely administered. Antiemetic agents such as ondansetron were used as needed. Bedside nursing staff assessed the pain scale every 15 min while patients were in the PACU, then every hour for the first 10 h, and every 3 h thereafter.

All statistical analyses were conducted using SAS version 9.4 (SAS Institute, Cary, NC). Univariate analyses were performed using a 2-sample *t* test or Wilcoxon rank-sum test for continuous variables and chi-square test or Fisher exact test for categorical variables. Patient-related risk factors that were statistically significant (*P* < 0.05) and baseline intraoperative narcotic dose were included in multivariable analyses. Differences in the pain scores were evaluated using mixed linear regression and repeated ANOVA. Bonferroni’s or Dunnett’s method was used for multiple comparisons as appropriate.

## RESULTS

### Demographics and Baseline Values

During the study period, 72 donors underwent the TAP/RS block (38 single-shot and 34 continuous), whereas 86 did not. The baseline characteristics were comparable between the groups except for body weight and body mass index. Owing to the multicollinearity of these variables, we adjusted only body weight in the analyses. Although the duration of surgery was not different, the intraoperative opioid doses were significantly different (Table 1).

**TABLE 1.** Univariable analysis of patient demographic

Variable	Block (n = 72)	Control (n = 86)	*P*
TAP (%)			
Bilateral	55 (76)	0	**<0.0001**
Continuous	34 (47)	0	
Sex, female (%)	52 (72)	57 (66)	0.42
Age (y), mean ± SD	41.0 ± 12.2	41.8 ± 11.8	0.66
Height (cm), mean ± SD	167.0 ± 9.7	168.6 ± 11.4	0.37
Weight (kg), mean ± SD	71.8 ± 13.3	77.8 ± 17.3	**0.01**
Body mass index (kg/m^2^), mean ± SD	25.7 ± 3.8	27.2 ± 4.1	**0.02**
Operative time (min), median (IQR)	141 (132–159)	137 (125–152)	0.59
Kidney laterality, left (%)	67 (93)	79 (92)	0.78
Intraoperative morphine equivalent dose (mg), median (IQR)			
	27.17 (20.5–31.7)	31.67 (25.0–38.3)	**0.001**
Per kg bodyweight	0.3710 (0.2993–0.4686)	0.4137 (0.3304–0.5325)	**0.017**

IQR, interquartile range; TAP, transverse abdominis plane.

### Postoperative Opioid Requirement

There were no statistically significant differences between the block and control groups in postoperative opioid requirements for the 6- and 24-h postoperative periods (Table 2). The median MME for the block group versus control group were 0.1290 versus 0.1095 mg/kg (*P* = 0.47) and 0.0318 versus 0.0230 mg/kg (*P* = 0.78) for the 0–6 and 6–24 h postoperative periods, respectively. These results remained consistent when the analysis was adjusted for the intraoperative opioid dose. The median MME did not differ by the initial infusion dose of ropivacaine; 0.1047 mg/kg was required for patients receiving <100 mg, whereas 0.1528 mg/kg was required for patients receiving >100 mg for the 6-h postoperative period (*P* = 0.24). This result was similar for the 6–24 h postoperative period: 0.030 versus 0.049 mg/kg (*P* = 0.38).

**TABLE 2.** Univariable analysis of postoperative narcotics, nonnarcotics, and adjunct medication requirement

Variable	Block (n = 72)	Control (n = 86)	*P*
Postoperative morphine equivalent dose (mg/kg bodyweight), median (IQR)			
0–6 h postoperative	0.1290 (0.0364–0.2440)	0.1095 (0.0438–0.2120)	0.47
6–24 h postoperative	0.0318 (0–0.0769)	0.0230 (0–0.0569)	0.78
24–48 h postoperative	0.0403 (0–0.1218)	0.0224 (0–0.0583)	0.06
Total nonnarcotic doses 0–48 h postoperative, median (IQR)			
Ketorolac (mg)	135 (120–135)	135 (120–150)	0.15
Acetaminophen (mg)	6000 (4650–7000)	5650 (4000–7000)	0.63
Total antiemetic doses 0–48 h postoperative, median (IQR)			
Ondansetron (mg)	8.0 (4.0–12.0)	4.0 (4.0–12.0)	0.36
Metoclopramide (mg)	0 (0–0)	0 (0–0)	0.10
Scopolamine (mg)	0 (0–0)	0 (0–0)	0.53
Promethazine (mg)	0 (0–0)	0 (0–0)	0.14

IQR, interquartile range.

Subgroup analyses comparing single-shot or continuous-block donors with control donors also showed no differences. The single-injection donors tended to require more opioids than the control group after 24 h, but this difference was not significant after correcting the significance level for multiple comparisons (data not shown).

### Pain Scale

**FIGURE 1.**
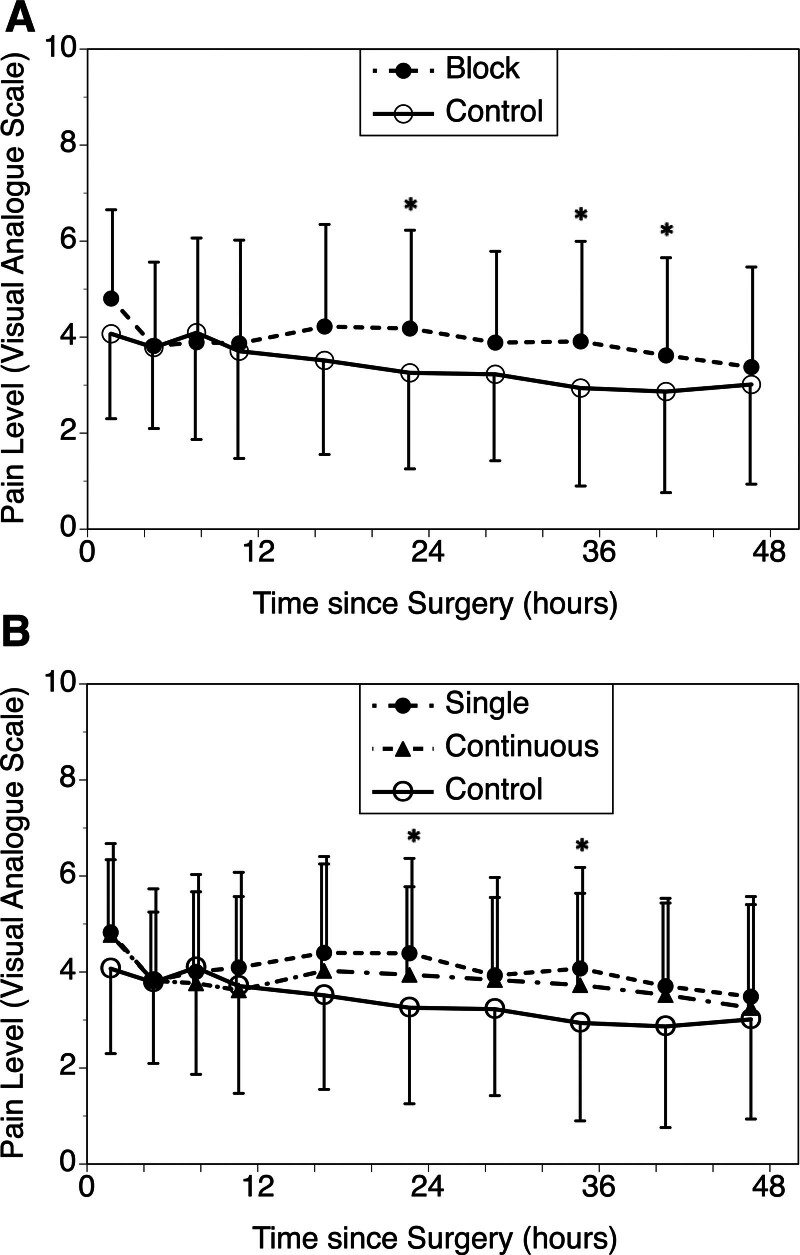
Post-operative pain level for the block group and control group. A, Postoperative VAS comparing the patients who received the TAP/RS block and patients who did not receive the block. Overall, the block group had higher VAS values than the control group. After adjusting the baseline values, there was no significant difference in overall VAS values. There were differences (*) between the groups at hours 18–24, 30–36, and 36–42 by the general linear model. However, these differences were not statistically significant when multiple comparisons were corrected. B, Subgroup analysis comparing the single-shot or continuous block to control patients. After adjusting the baseline values, overall VAS values were similar (*P* = 0.449). There were differences (*P < 0.05 before correction) between the single-shot and the control groups at hours 18–24 and 30–36. However, these differences were not statistically significant when multiple comparisons were corrected. RS, rectus sheath; TAP, transverse abdominis plane; VAS, Visual Analog Pain Scale.

The pain scale over the 48-h postoperative period is shown in Figure 1. Baseline pain scales differed significantly (control: 4.07 ± 1.90 versus block: 4.80 ± 1.71; *P* = 0.0149), and the block donors continued to have higher pain scales afterward. When baseline values were adjusted, the differences between the groups decreased, but no additional pain reduction was observed with the block (*P* = 0.242). Subgroup analysis showed that the pain scales of single-shot and continuous-block donors were similar to those of control donors (*P* = 0.449).

### Length of Stay

The postoperative LOS was statistically significantly longer in the block group (2.18 d [2.1–2.7 d] versus 2.13 d [2.1–2.2 d]; *P* = 0.0122), but the difference (1.2 h) was not clinically significant. The patients who received TAP spent significantly longer post-surgically in the operating room or PACU; the median time between the skin closure and PACU discharge was 183 versus 148 min (*P* < 0.0001), likely due to the block procedure time.

## DISCUSSION

This retrospective, single-center study of patients undergoing HALN demonstrated the limited analgesic effectiveness of the TAP block. When around-the-clock ketorolac and acetaminophen were used, neither postoperative single-shot nor continuous TAP block decreased the postoperative opioid requirement or improved pain scores.

The ERAS model has been adapted to many surgical procedures and a shift to opioid-sparing analgesia has improved postoperative patient outcomes. A systematic review of ERAS protocols for living kidney donors presented a limited number of randomized controlled trials; most were retrospective cohort studies.^15^ Although the positive benefits of ERAS were suggested in the review, only a few studies have investigated the effectiveness of the TAP block. One retrospective single-center study discovered that bupivacaine or liposomal bupivacaine TAP block reduced the postoperative opioid requirement after laparoscopic donor nephrectomy.^16^ Another small study examined the preoperative injection of a TAP block for robotic-assisted donor nephrectomy.^17^ They found that using liposomal bupivacaine TAP in the context of ERAS reduced LOS and opioid requirements. A larger single-center retrospective study evaluated patient outcomes before and after implementation of the ERAS protocol, including preoperative TAP block.^18^ Although pain scores remained similar, overall narcotic use and LOS were significantly reduced after the initiation of the protocol.

At least 2 randomized control studies have reported the benefits of bilateral TAP blocks. A trial by Aniskevich et al^19^ compared ropivacaine TAP administered preoperatively with saline in patients undergoing elective living-donor nephrectomy or single-sided nephrectomy for tumor. The researchers found that TAP blocks reduced overall pain scores at 24 h, with a trend toward decreased narcotic consumption. Nausea, vomiting, sedation, and LOS were not significantly different between the 2 study groups. Similarly, a randomized control trial by Hosgood et al^20^ compared preoperative single-injection bupivacaine and saline TAP block. They found that bupivacaine provided better short-term outcomes (ie, less narcotic use and less pain), while total narcotic use and LOS were similar.

Two significant differences exist between these studies showing the benefits of TAP blocks and the current study. First, our control patients received around-the-clock ketorolac and acetaminophen, whereas the pre-ERAS (control) patients of prior ERAS studies did not receive such analgesics. Although nonsteroidal anti-inflammatory drug use is generally associated with the risk of kidney dysfunction, perioperative ketorolac use is widely accepted in live-donor nephrectomy and is considered to be safe.^21-23^ However, 1 study suggested a possible negative effect of ketorolac on 1-y kidney function after donor nephrectomy.^24^ As the number of older living kidney donors increases, the use of ketorolac may be associated with worsening kidney function in some donors. The current results might have differed if we had compared TAP and non-TAP block patients without ketorolac use.

Second, our patients received TAP after surgery due to logistical constraints, whereas many TAP blocks in previous studies were performed before surgery. The effectiveness of TAP blocks performed preoperatively and postoperatively varies, suggesting that results depend on the local anesthetic used, the type and duration of surgery, and concomitant medications.^25-28^ For long-duration surgeries, the effect of a TAP block with short-acting anesthetics may not persist postoperatively. Conversely, utilizing long-acting anesthetics in a preoperative TAP block may be most beneficial for shorter operations. Nevertheless, in our study, the benefits of the TAP block might have been more pronounced had it been administered preoperatively, particularly with continuous infusion. Although our block patients received less intraoperative narcotic dose, this was not the direct effect of the TAP block since the block was performed after the surgery. Some of our patients received continuous TAP infusion through a catheter; however, we did not observe any additional analgesic benefit over a single injection of ropivacaine. The results of our subgroup analyses were consistent with those of Yeap et al.^29^ They found that a continuous TAP block delivered after skin closure did not provide additional pain relief when intravenous patient-controlled analgesia for breakthrough pain was used.

This study has some limitations owing to its retrospective nature. Selection bias may have occurred because of nonrandomization; patients who received the TAP block may have had a lower tolerance for pain or may have been more sensitive to the experience of pain. However, we did not observe any differences after adjusting for the baseline pain scores. Furthermore, to compensate for the lack of a regional block, anesthesiologists may have administered more intraoperative narcotics to patients who did not consent to the TAP block. Regardless, the total perioperative and early postoperative narcotic doses were similar, and no effect of the TAP block was observed.

In conclusion, this retrospective study demonstrates that despite the abovementioned limitations, postoperative single-shot or continuous TAP block has limited utility when around-the-clock ketorolac and acetaminophen are used. Future randomized clinical trials should be conducted to evaluate the adjunctive effect of TAP block when around-the-clock ketorolac and acetaminophen are used for HALN.

## ACKNOWLEDGMENTS

The authors thank the Clinical and Translational Science Center at UC Davis for biostatistical assistance.
